# Effects of Extremely Low Frequency Electromagnetic Fields on Melanogenesis through p-ERK and p-SAPK/JNK Pathways in Human Melanocytes

**DOI:** 10.3390/ijms18102120

**Published:** 2017-10-11

**Authors:** Yu-Mi Kim, Sang-Eun Cho, Soo-Chan Kim, Hyun-Joon Jang, Young-Kwon Seo

**Affiliations:** 1 Department of Medical Biotechnology (BK21 Plus team), Dongguk University, Goyang-si 10326, Korea; kjmtik@nate.com (Y.-M.K.); ssasng41@naver.com (S.-E.C.); filter4u1@naver.com (H.-J.J.); 2 Department of Electric and Electrical Engineering, Institute for Information Technology Convergence, Hankyong National University, Anseong-si 17579, Korea; sckim@hknu.ac.kr

**Keywords:** extremely low frequency electromagnetic fields (ELF-EMFs), melanogenesis, MITF, p-CREB, tyrosinase

## Abstract

This study evaluated frequency-dependent effects of extremely low frequency electromagnetic fields (ELF-EMFs) on melanogenesis by melanocytes in vitro. Melanocytes were exposed to 2 mT EMFs at 30–75 Hz for 3 days before melanogenesis was examined. Exposure to ELF-EMFs at 50 and 60 Hz induced melanogenic maturation without cell damage, without changing cell proliferation and mitochondrial activity. Melanin content and tyrosinase activity of cells exposed to 50 Hz were higher than in controls, and mRNA expression of tyrosinase-related protein-2 was elevated relative to controls at 50 Hz. Phosphorylated cyclic adenosine monophosphate response element-binding protein (p-CREB) levels were higher than controls in cells exposed to ELF-EMFs at 50–75 Hz. Immunohistochemical staining showed that melanocyte-specific markers (HMB45, Melan-A) were strongly expressed in cells exposed to EMFs at 50 and 60 Hz compared to controls. Thus, exposure to ELF-EMFs at 50 Hz could stimulate melanogenesis in melanocytes, through activation of p-CREB and p-p38 and inhibition of phosphorylated extracellular signal-regulated protein kinase and phosphorylated stress-activated protein kinase/c-Jun N-terminal kinase. The results may form the basis of an appropriate anti-gray hair treatment or be applied in a therapeutic device for inducing repigmentation in the skin of vitiligo patients.

## 1. Introduction

Melanocytes, which differentiate from melanoblasts, can produce the pigment melanin in a process called melanogenesis. Melanin is present in tissues, such as skin and hair, and in the eyes, and it is synthesized by the melanosome organelle within the melanocyte [1]. Melanin can block a certain amount of ultraviolet radiation (UVR), protecting the skin, and scavenge free oxygen radicals [2]. 

The skin contains a local defensive melanocortin system to neutralize a wide range of external noxious stimuli (principally UVR) and consists of the pigment melanin and its associated cleaved proopiomelanocortin (POMC) peptides. In cutaneous melanocytes, UVR stimulates POMC formation, with resultant release of several POMC peptides via differential enzymatic cleavage of *POMC* by prohormone convertases. Consequently, corticotropin (ACTH), and α-, β-, and γ-melanocyte-stimulating hormones (MSHs) are produced that can influence melanogenesis. Melanin pigment synthesized in this way can act as a buffer molecule to antagonize the noxious effects of physical, biological, and chemical insults [3,4].

The biosynthesis of melanin is a complicated process involving many factors. The important positive regulators of melanin pigmentation include endothelins, histamine, and eicosanoids, acting via interaction with cell surface receptors. Melanocortin-1 receptor (MC1R) is expressed in melanocytes and activation of MCIR by its ligand α-MSH, plays a crucial role in pigmentation by regulating the intracellular levels of cyclic adenosine monophosphate (cAMP) [5]. Besides serving as substrates and intermediates of melanogenesis, it is well known that l-tyrosine and l-dihydroxyphenylalanine (l-DOPA) are also bioregulatory agents, acting not only as inducers and positive regulators of melanogenesis but also as regulators of other cellular functions. Tyrosinase can act both as a regulatory protein by interacting with other proteins and as a regulator of intra- and extracellular concentrations of biologically active molecules (l-tyrosine and l-DOPA). l-DOPA can also regulate cell functions and cellular metabolism through non-receptor-mediated processes that act directly or through intermediates of melanogenesis generated by its non-enzymatic or enzymatic oxidation [6].

Hair follicular melanogenesis involves, sequentially, the melanogenic activity of follicular melanocytes, the transfer of melanin granules into cortical and medulla keratinocytes, and the formation of pigmented hair shafts [7]. The common aging process of graying hair is caused by defects in or the frailty of melanocytes [8]. There are also several associated diseases, such as vitiligo, that occur when melanin synthesis is impaired because the melanocytes are either absent or no longer function [9]. Many possible causes of vitiligo, including immunologic, genetic, stress, and neural mechanisms, and biochemical factors [10] have been proposed, but the etiopathogenesis of the disease is still enigmatic. However, it is believed that the disease is mainly a result of the destruction of melanocytes and obstruction of the melanin synthesis pathway [11].

Many physical treatment methods have been proposed to increase melanin synthesis in the melanocytes of vitiligo patients. Narrowband ultraviolet B (NB-UVB) phototherapy (311–313 nm peak emission) has become a popular treatment option for generalized vitiligo [12]. Significant repigmentation has been reported in a majority of patients with some follicular repigmentation in the remaining 34%, after treating vitiligo patients with a low energy HeNe laser (632 nm, 25 mW/cm^2^). Consequently, it has been suggested that low level laser light therapy (LLLT) and excimer laser (308 nm) light may provide an effective alternative treatment for vitiligo patients [13]. These types of phototherapy can increase repigmentation. The laser stimulates melanocyte proliferation through enhanced α_2_β_1_ integrin expression and induces melanocyte growth through upregulation of phosphorylated cAMP response element-binding protein (p-CREB) [14]. Thus, LLLT treatment activates melanoblasts, melanocytes, and other cells, inducing or enhancing melanin synthesis.

Depending on cell type, various stimulation techniques have been used to activate cells, including cyclic pressure, cyclic compressive load, uniaxial strain, perfusion, shear and compression, ultrasound, laser, electrical stimulation, and electromagnetic field, for instance. Physical stimulation, in particular, has been extensively investigated and numerous stimulation devices have been designed and are in clinical use for raising cellular activity levels.

Low frequency low energy pulsed electromagnetic fields (PEMFs) have recently become a research focus to achieve cell activation, proliferation, and differentiation [15]. Exposure to extremely low frequency electromagnetic fields (ELF-EMFs) has been shown to modulate the osteogenic differentiation of human bone marrow-multipotent stromal cells (hBM-MSCs) and was associated with increased alkaline phosphatase (ALP) levels and higher expression of osteogenesis-related genes in hBM-MSCs [16,17]. EMF application has also been proposed to treat bone fractures and has been used to slow bone matrix loss in animal experiments [18,19]. Several studies have demonstrated that EMFs enhance neuronal differentiation and cellular and molecular processes, such as neural marker expression [20,21,22].

Melanoblasts are neural crest-derived precursors of melanocytes, with the ability to produce melanin. They migrate to various parts of the body during the early stages of embryonic development before they become mature melanocytes. We hypothesize that EMFs may also activate melanocytes and increase melanogenesis, and the present study investigated frequency-dependent effects of EMFs on melanogenesis. Melanocytes were exposed to 30–75 Hz, 2 mT EMFs for 3 days. Melanogenesis was measured in terms of melanin synthesis and tyrosinase activity, by western blotting, reverse transcription real-time quantitative polymerase chain reaction (RT-qPCR), and immunohistochemical staining.

## 2. Result

### 2.1. Cell Proliferation and Mitochondrial Activity

From the melanocyte strategic location in the basal layer of the epidermis, melanocytes communicate via their dendritic processes with 36–40 keratinocytes, to whom they transfer melanin-containing melanosomes [23]. Thus, the dendritic process is pivotal for skin melanocytes. 

**Figure 1:**
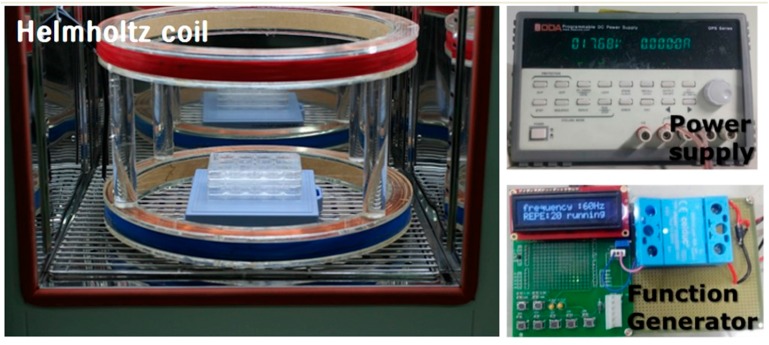
Photograph of the electromagnetic field (EMF) device. Pulsed EMF was generated using a pair of Helmholtz coils of 40 cm diameter and 15 cm separation.

We used Helmholtz coils, comprising two identical circular coils (diameter = 40 cm, distance = 15 cm), and oriented to produce a region of nearly uniform rectangular pulse EMF (Bm = 2 mT). The coils operated on alternating current, generating the PEMF. The coil current was controlled by a generator. The system was supplied by COMSOL 3.4 (COSMOL, Burlington, MA, USA) and a Tesla TM-701 meter (KANETEC, Tokyo, Japan) for monitoring magnetic flux density distribution. The culture dishes, multi-well plates, and multi-well plate cover slides were located in the center of the Helmholtz coils, as shown in Figure 1.

**Figure 2:**
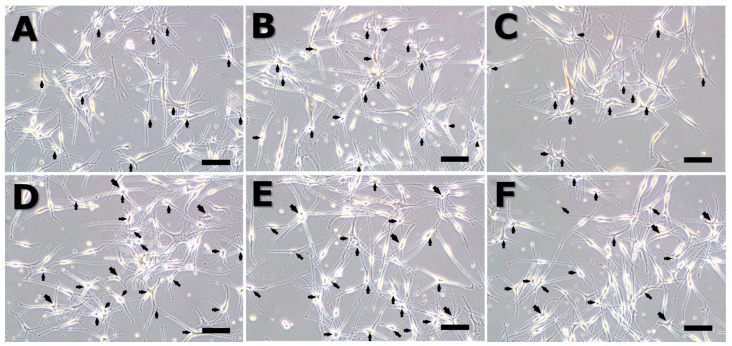
Melanocyte morphology after stimulation by an extremely low frequency electromagnetic field (ELF-EMF) for 72 h. All groups were cultured in M254 media. Before ELF-EMF exposure, cells were cultured in modified M254 media containing forskolin, melanocyte-stimulating hormone (α-MSH), and 12-*O*-tetradecanoyl-phorbol-13-acetate for 72 h. After culturing in differentiation media, cells were treated with continuous ELF-EMFs. The α-MSH group was cultured in M254 media added to 5 nM α-MSH. (**A**) control; (**B**) α-MSH; (**C**) 30 Hz; (**D**) 50 Hz; (**E**) 60 Hz; and (**F**) 75 Hz. Original magnification was ×100; bar = 100 μm.

Figure 2 shows the morphology of the melanocytes after 72 h. Cells grown under control conditions and cells exposed to EMFs at 75 Hz showed characteristic bipolar dendritic processes, whereas cells exposed to EMFs at 50 and 60 Hz displayed highly branched dendritic networks and multipolar morphology (arrow). The number of dendritic networks and multipolar morphology was scored from observing a microscopy image (Table 1). Thus, exposure to ELF-EMFs at 50 and 60 Hz induced melanogenic maturation in the absence of chemical agents but did not trigger apoptosis or necrosis.

**Figure 3:**
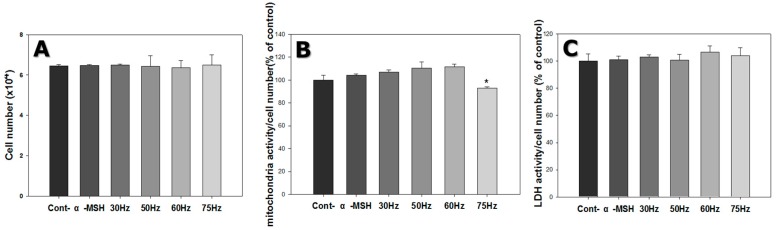
Cell proliferation, mitochondrial activity, and cytotoxicity of melanocytes after 72 h exposure to extremely low frequency electromagnetic fields. (**A**) Cell count (to measure cell proliferation); (**B**) 3-(4,5-dimethylthiazol-2-yl)-2,5-diphenyltetrazolium bromide assay (to determine cell mitochondrial activity); (**C**) Lactate dehydrogenase (LDH) assay (to examine cellular damage). Each bar represents mean ± standard error from independent experiments performed in triplicate (*n* = 5). * *p* < 0.05, compared to the control.

Cell count was approximately 6.4 × 10^4^ for cells grown under control conditions, 6.5 × 10^4^ for cells exposed to α-MSH only, 6.4 × 10^4^ for cells exposed to 30 Hz, 6.5 × 10^4^ for cells exposed to 50 Hz, 6.4 × 10^4^ for cells exposed to 60 Hz, and 6.5 × 10^4^ for cells exposed to 75 Hz. Thus, ELF-EMF exposure did not significantly affect cell proliferation (Figure 3A). 

The mitochondrial activity of the cells was measured by the MTT assay. This method is based on the conversion of MTT into formazan crystals by living cells, which determines mitochondrial activity. Mitochondrial activity was evaluated by dividing the cell number by the measured mitochondrial activity. Mitochondrial activity per cell was similar for all ELF-EMF exposure cases, for most of the exposed group compared to the control (Figure 3B).

### 2.2. Cytotoxicity

LDH is a cytoplasmic enzyme that catalyzes the reversible interconversion between pyruvic and lactic acid and is released when the cell membrane is damaged [24]. Hence, LDH is a convenient indicator of cellular injury or stress. Media were collected and analyzed after 3 days, to assess the condition of the cells. LDH activity was evaluated by dividing by the cell count (Figure 3C) and was similar for the 30, 50, 60 and 75 Hz groups compared to the control. Based on the LDH results in Figure 3C, the ELF-EMFs did not influence the damage to cell membranes.

### 2.3. Melanin Content

**Figure 4:**
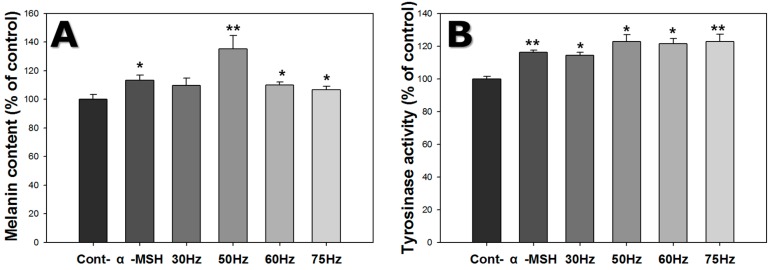
Effect of extremely low frequency electromagnetic fields (ELF-EMFs) on melanogenesis of melanocytes after exposed to ELF-EMFs for 72 h. After ELF-EMFs exposure, melanin content and tyrosinase activity were determined. (**A**) Melanin content was detected by the melanin content assay; (**B**) Tyrosinase activity was measured by the tyrosinase assay. Each bar represents the mean ± standard error of independent experiments performed in triplicate (*n* = 3). * *p* < 0.05, ** *p* < 0.01, compared to the control.

The amount of melanin in melanocytes was investigated by the melanin content assay, as shown in Figure 4A. Melanin content increased 1.1-fold on average, for cells exposed to ELF-EMFs. Melanin content of cells exposed to ELF-EMF at 50 Hz was 1.3 times the controls, and 1.1 times the cells exposed to α-MSH only. Thus, ELF-EMFs induced increased melanogenesis.

### 2.4. Tyrosinase Activity

Melanocyte tyrosinase activity was assessed using the tyrosinase activity assay, as shown in Figure 4B. Tyrosinase activity increased in cells exposed to ELF-EMFs. Cells exposed to 50 and 75 Hz were 1.2 times the controls, and 1.1 times those exposed to α-MSH only. Therefore, ELF-EMF exposure increased tyrosinase activity.

### 2.5. RT-qPCR

**Figure 5:**
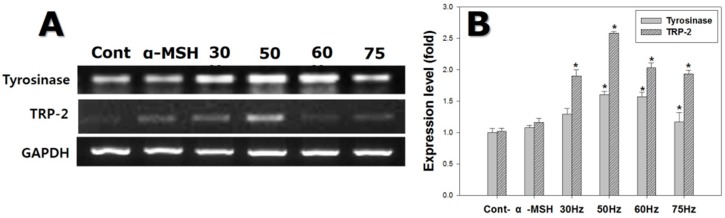
Gene expression detected by reverse transcription polymerase chain reaction (RT-PCR) on melanocytes after exposure to extremely low frequency electromagnetic fields for 72 h. (**A**) Electrophoretic RT-PCR for melanogenesis-related genes; (**B**) mRNA expression of melanogenesis-related genes, using glyceraldehyde-3-phosphate dehydrogenase (*GAPDH*) as the reference protein. Each bar represents the mean ± standard error of independent experiments performed in triplicate (*n* = 5). * *p* < 0.05, compared to the control (Cont).

The mRNA expression levels of key melanogenesis-related genes, such as tyrosinase and *TRP-2*, were measured at 72 h, as shown in Figure 5. Tyrosinase mRNA levels in cells exposed to ELF-EMFs were on average 1.3-fold those in the controls. In particular, cells exposed to ELF-EMFs at 50 and 60 Hz showed tyrosinase expression 1.5 times the controls.

TRP-2 mRNA levels in cells exposed to ELF-EMFs were on average 2.0 times the controls. Cells exposed to 50 Hz showed elevated *TRP-2* expression, at 2.5 times the controls. Consequently, ELF-EMF exposure stimulated the expression of melanogenesis-related mRNA species.

### 2.6. Western Blotting

**Figure 6:**
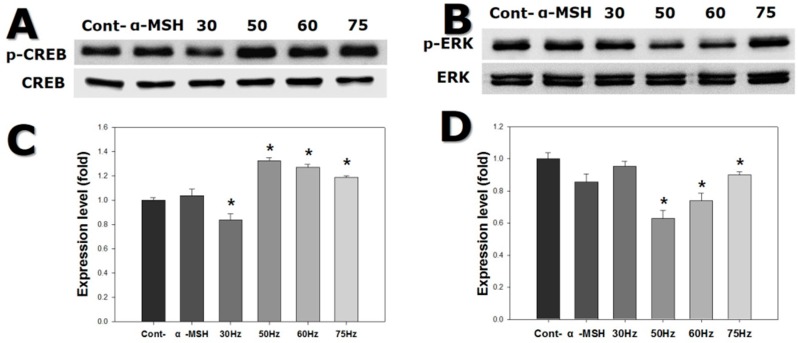
Western blot of cyclic adenosine monophosphate response element-binding protein (CREB) and extracellular signal-regulated kinase (ERK) expression in melanocytes after exposure to extremely low frequency electromagnetic fields for 72 h. (**A**) CREB and phosphorylated-CREB (p-CREB) banding; (**B**) p-CREB expression; (**C**) ERK and phosphorylated ERK (p-ERK) expression; (**D**) p-ERK expression. Each bar represents the mean ± standard error of independent experiments performed in triplicate (*n* = 5). * *p* < 0.05, compared to the control (Cont).

**Figure 7:**
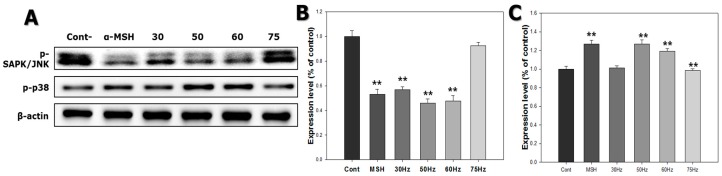
Western blot of stress-activated protein kinase/c-Jun N-terminal (SAPK/JNK) and p38 expression in melanocytes after exposure to extremely low frequency electromagnetic fields for 72 h. (**A**) Phosphorylated-SAPK (p-SAPK)/JNK and phosphorylated-38 (p-p38) banding; (**B**) p-SAPK/JNK expression; (**C**) p-p38 expression. Each bar represents the mean ± standard error of independent experiments performed in triplicate (*n* = 3). ** *p* < 0.01, compared to the control (Cont).

In order to study processes related to melanogenesis in melanocytes exposed to ELF-EMFs, we assessed the activation of p-CREB and mitogen-activated protein kinases (MAPKs) signaling. CREB transcription factor p38 is involved in upregulation, while ERK and JNK pathways are involved in downregulation of melanogenesis. Figure 6 shows that p-CREB levels increased 1.2-fold in cells exposed to ELF-EMFs. More specifically, cells exposed to 50 and 75 Hz displayed high levels of p-CREB. p-ERK activation was reduced in cells exposed to ELF-EMFs, particularly those exposed to 50 and 60 Hz. p-SAPK/JNK activation was also decreased in cells exposed to ELF-EMFs, mainly for cells exposed to 50 and 60 Hz. Conversely, *p-p38* activation was increased in cells exposed to ELF-EMFs, principally for the cells exposed to 50 and 60 Hz (Figure 7). Thus, ELF-EMF exposure stimulated melanogenesis.

**Figure 8:**
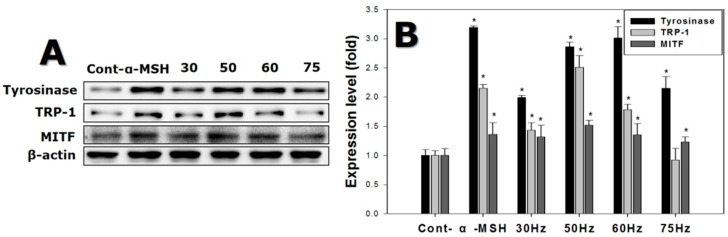
Western blot of protein expression levels detected in melanocytes after exposure to extremely low frequency electromagnetic fields for 72 h. (**A**) Melanogenesis-related protein banding, using β-actin as an internal control; (**B**) Melanogenesis-related protein expression. Each bar represents the mean ± standard error of independent experiments performed in triplicate (*n* = 5). * *p* < 0.05, compared to the control (Cont).

Melanogenesis in melanocytes exposed to ELF-EMFs for 72 h, was further investigated by quantifying the melanogenesis-related proteins after the exposure, with western blotting. Figure 8 shows that tyrosinase expression levels increased on average 2.0-fold in cells exposed to ELF-EMFs. Cells exposed to 50 and 60 Hz had strong tyrosinase upregulation compared to the controls.

TRP-1 expression levels were 2.5 times higher for cells exposed to ELF-EMFs than the control. Finally, cells exposed to 50 and 60 Hz, expressed *MITF* more strongly, exhibiting expression levels 1.5 times greater than the controls.

### 2.7. Immunohistochemistry

**Figure 9:**
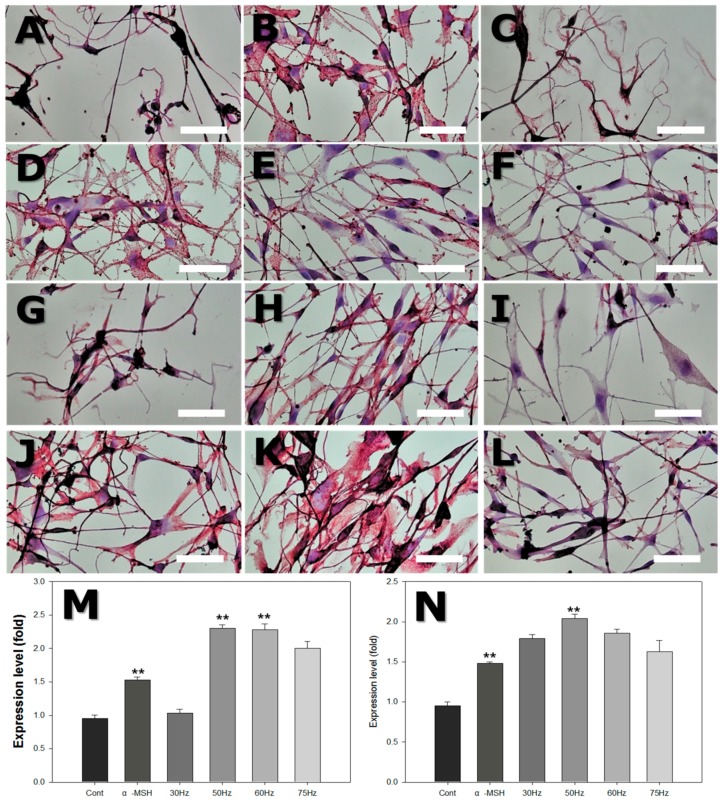
Light microscope photograph of human skin melanocytes attached to dish surface, stained with HMB45 and Melan-A after exposure to extremely low frequency electromagnetic field (ELF-EMF) for 72 h. (**A**–**F**) with HMB45 staining: (**A**) Control (Cont); (**B**) alpha-melanocyte-stimulating hormone (α-MSH); (**C**) 30 Hz; (**D**) 50 Hz; (**E**) 60 Hz; and (**F**) 75 Hz. (**G**–**L**) with Melan-A staining: (**G**) Control; (**H**) α-MSH; (**I**) 30 Hz; (**J**) 50 Hz; (**K**) 60 Hz; and (**L**) 75 Hz. Original magnification was ×400; bar = 50 μm. Quantitative analysis of expression levels of HMB45 (**M**) and Melan-A (**N**) after ELF-EMFs exposure. Each bar represents the mean ± standard error of independent experiments performed in triplicate (*n* = 4). ** *p* < 0.01, compared to the control.

Immunohistochemical staining was performed to assess the expression of proteinaceous markers of melanogenesis. HMB45 is a melanosome-specific antigen, and Melan-A is a melanocyte differentiation antigen. The relative staining intensity was evaluated by light microscopy, as shown in Table 2 and Figure 9. Compared to controls, both melanocyte-specific markers (HMB45, Melan-A) were strongly expressed in cells exposed to ELF-EMFs at 50 and 60 Hz.

## 3. Discussion

Melanin synthesis and pigment transfer to bulb keratinocytes are dependent on the availability of melanin precursors and regulation by signal transduction pathways intrinsic to skin and hair follicles, which are both receptor-dependent and -independent, act through auto-, para- or intracrine mechanisms and can be modified by hormonal. Some investigators have documented that signal transduction pathways involving SCF/c-Kit and ET1, ET3/ETA, and ETB play a crucial role in normal follicle melanogenesis. Also, many other researchers revealed that pro-pigmentary activity is initiated by binding of POMC-derived ACTH, α-MSH, and β-MSH peptides. Particularly the β-endorphin/μ-opiate receptor system also participates in the regulation of both human epidermal and follicular melanocyte biology, by inducing changes in dendricity, proliferation, and melanogenesis. Furthermore, it is well established that melatonin, interleukin-1 and -6, tumor necrosis factor-α, transforming growth factor-β, interferon-γ, glucocorticoids, triiodothyronine, and dopaminergic and cholinergic agonists negatively regulate melanogenesis [7].

ELF-EMF interactions with biological systems and resultant health effects have been investigated, suggesting influences on numerous cell types and processes, including cell migration, differentiation, apoptosis, and stress responses [25,26]. Moreover, ELF-EMFs have been investigated regarding osteogenesis, bone healing, and neurogenesis, and the current authors have previously reported that ELF-EMFs promote neural differentiation of mesenchymal stem cells [27]. These above-mentioned studies suggest that ELF-EMF exposure affects cells through physical or mechanical effects on both intracellular and membrane proteins, including ion channels, membrane receptors, and enzymes [28]. However, previous research has not attempted to use ELF-EMFs to stimulate melanin synthesis in melanocytes.

The main purpose of this study was to confirm the effect of ELF-EMFs on melanogenesis and identify optimal conditions for ELF-EMF-induced melanogenesis in melanocytes. Frequency-dependent cytotoxicity of EMFs involved examination of cell morphology and the LDH assay results, which confirmed that the EMFs used in this study were not cytotoxic to melanocytes.

Melanocytes reside in the basal layer and produce the protective melanin pigment that is transferred to neighboring keratinocytes [4]. Melanin is synthesized by conversion of tyrosine into dopaquinone, catalyzed by tyrosinase, and the tyrosinase-related proteins TRP-1 and TRP-2 are involved in this process. Therefore, to investigate the effects of EMFs on melanogenesis, we studied the activity of tyrosinase and the gene and protein expression of tyrosinase, TRP-1, and TRP-2. Tyrosinase is the key enzyme in melanin synthesis. TRP-1 and TRP-2 share 40–45% amino acid identity with tyrosinase and are, likewise, critical for melanogenesis, functioning as downstream enzymes in the biosynthetic pathway [29,30]. Increased tyrosinase activity in melanocytes could be achieved by stimulation of tyrosinase gene expression, leading to higher levels of the corresponding protein in melanocytes.

Melanocyte tyrosinase activity increased after exposure to EMFs at 50–75 Hz, as shown in Figure 4B, and PCR and western blot analysis confirmed that the expression of tyrosinase increased at both the gene and protein levels, after exposure to 50 and 60 Hz. The expression of TRP-2 was significantly increased at 50 Hz (Figure 5 and Figure 6).

The MITF transcription factor is a key regulator of melanocyte development, function, and survival by regulating genes involved in the cell cycle and melanocyte differentiation [31]. Activated MITF also raises protein levels, including tyrosinase, dopachrome tautomerase, TRP-1, as well as pigment production [32,33,34].

We investigated MITF protein expression level using western blotting. MITF protein expression increased with exposure to ELF-EMF at 50–60 Hz, which correlated with tyrosinase and TRP-1 expression. A previous study reported increased MITF and tyrosinase expression from physical stimulation of melanocytes [35]. The authors of that study demonstrated the effects of NB-UVB on the maturation of the melanocyte lineage differentiated from hair follicle-derived neural crest stem cells in vitro. NB-UVB increased the expression of MITF and tyrosinase during melanocytic differentiation and is currently used in clinical vitiligo phototherapy.

CREB is a basic leucine zipper (bZip) transcription factor, and p-CREB increases MITF expression [36]. Previously, the influence of aromatic-turmerone on inhibiting melanogenesis was suppressed by CREB activation, and expression of MITF, tyrosinase, TRP-1, and TRP-2 in B16F10 melanoma cells [37]. In contrast, it was reported that ginsenoside-induced melanogenesis, upregulated expression of MITF, tyrosinase, and p-CREB in human melanocytes [38]. Thus, increases in p-CREB expression are believed to increase melanogenesis. In the present study, western blotting analysis verified that expression of p-CREB stimulated melanogenesis in human melanocytes exposed to ELF-EMFs.

The ERK pathway is a key signaling pathway involved in controlling cell growth. ERK activation also increases cell proliferation in a variety of cell types [39]. The ERK pathway is a major signaling cascade, participating in many signal transduction processes, and is involved in regulating MITF [40]. ERK activation leads to phosphorylation of MITF, which results in decreased melanin synthesis by melanocytes [37]. ERK is one of the MAPK family, which includes JNK and p38.

Similar to ERK activation, SAPK/JNK activation is also related to downregulation of melanogenesis. In contrast, p38 activation increases melanogenesis by stimulating expression of MITF and tyrosinase transcription [41,42,43]. Shogaol inhibits α-MSH-induced melanogenesis by accelerating the ERK and PI3k/serine-threonine kinase (Akt) pathways in B16F10 melanoma cells [44] and was also found to downregulate MITF expression. Ascorbic acid stimulates melanogenesis in B16F10 murine melanoma cells by expression of MITF, tyrosinase, TRP-1, and TRP-2, as well as activation of the MAPK family, accelerates p38 activation and suppresses activation of JNK and ERK [41]. Consequently, increased p-ERK and p-SAPK/JNK expression are believed to decrease melanogenesis, whereas p-ERK and p-SAPK/JNK downregulation increases melanogenesis. According to the western blotting analysis results of the current study, human melanocytes exposed to EMFs at 50 and 60 Hz, increased melanogenesis in association with p-ERK and p-SAPK/JNK downregulation and p38 upregulation.

HMB-45 is an antigen that appears in adult melanocytes during stimulation and in fetal skin, as well as in melanomas [45]. Melan-A has also been identified as a melanocytic differentiation marker, which is recognized as an antigen on melanoma cells, by cytotoxic T-lymphocytes [46]. In the current study, melanocytes stained strongly positively for HMB-45 and Melan-A, when exposed to α-MSH or ELF-EMFs at 50–60 Hz. Hence, ELF-EMFs at these frequencies can activate melanocytes and stimulate melanin synthesis.

In summary, the present study shows that melanogenesis can be stimulated by exposure to ELF-EMFs.

## 4. Materials and Methods

### 4.1. Cell Culture

QualiCell^®^ human melanoblast stem cells (Creative Bioarray, New York, NY, USA) were maintained in culture in Medium 254 (M254, Invitrogen, Waltham, MA, USA) with propidium monoazide-free human melanocyte growth supplement (HMGS-2, Invitrogen) in an incubator with 5% humidified atmosphere at 37 °C. Melanoblasts from passages 3 to 7 were used for experiments. The medium was changed every 2–3 days. For subculture, cells were washed with phosphate buffered saline (PBS), detached with Accutase (Innovative Cell Tech., San Diego, CA, USA), and passaged at a plate 1:3 plate ratio when the cells reached 80–90% confluence.

Melanoblasts were cultured in melanocyte differentiation medium, to produce melanocytes. The differentiation medium consisted of M254, HMGS-2, 10 nM α-MSH (Sigma Aldrich, St. Louise, MO, USA), 10 nM 12-*O*-tetradecanoyl-phorbol-13-acetate (TPA; Sigma Aldrich), and 20 μM forskolin (Sigma Aldrich). Experiments progressed once the melanoblasts had been treated with the differentiation medium for 3 days when the medium was replaced with M254 medium, and the cells were exposed to ELF-EMFs.

### 4.2. Exposure to Extremely Low Frequency Electromagnetic Fields (ELF-EMFs)

We used pulsed ELF-EMFs (2 mT, for 30, 50, 60, and 75 Hz) and the coils were placed in a 37 °C, 5% CO_2_ incubator. Cells were exposed to EMFs for 72 h. The stimulus waveform was pulse type, with 0.25 μs duration and 20 G (2 mT) intensity. Control cultures were located in a separate incubator to avoid ELF-EMF exposure, and the control media were identical to the experimental cell cultures. 

ELF-EMF exposure was performed in the following order. Melanoblasts were seeded in a 6-well plate or on a coverslip in a multi-well plate and cultured with melanocyte induction media for 3 days. The media were then changed to melanocyte culture media M254, and either exposed to the ELF-EMF or 5 nM MSH was added (control group).

### 4.3. Cell Proliferation Measurement and Mitochondrial Activity Assay

For cell proliferation and 3-(4,5-dimethylthiazol-2-yl)-2,5-diphenyltetrazolium bromide assay (MTT) assay, cells were seeded at 1 × 10^4^ cells per well in six-well plates and cultured with melanocyte differentiation media for 3 days. After induction of differentiation, ELF-EMF exposure was performed for 72 h.

A cell counter was used to measure cell proliferation (Scepter™, Millipore Corporation, Billerica, MA, USA) and cell activity was assessed by the MTT assay, a colorimetric assay for measuring cell metabolic activity.

For the MTT assay, each well was treated with MTT solution (5 mg/mL, Sigma Aldrich), and the plates were incubated at 37 °C for 1.5 h. The solution was then replaced with dimethyl sulfoxide (DMSO), to dissolve the formazan, and shaken for 5 min. The absorbance of the resulting solution was measured at 570 nm.

### 4.4. Cytotoxicity Assay

Cytotoxicity was evaluated by the lactate dehydrogenase (LDH) assay. For the LDH assay, cells were seeded at 1 × 10^4^ cells per well in six-well plates and cultured with melanocyte differentiation media for 3 days. After induction of differentiation, ELF-EMF exposure was performed for 72 h.

The cultured media was then collected, and an LDH-LQ kit was used (Asan Pharmaceutical, Seoul, Korea). The cultured medium (100 μL) was placed in a 96-well plate, 50 μL of working solution was added, and the plates then incubated at room temperature for 30 min. The reaction was terminated with 50 μL of stop solution (1 N HCl). Absorbance was measured at 490 nm.

### 4.5. Melanin Content Assay

The melanin content of cultured melanocytes exposed to ELF-EMFs was determined using a modified method of a previously published approach [47]. Cells were seeded at 1 × 10^4^ cells per well in six-well plates. After removing the culture medium from the plates, the cells were solubilized with 10% DMSO dissolved in 1 M NaOH and boiled at 80 °C for 2 h. Next, the cells were centrifuged at 15,000 rpm for 15 min and the supernatant extracted. The melanin content of the supernatant was measured using an enzyme-linked immunosorbent assay (ELISA) plate reader at 405 nm (Spectrum Analyzer. Victor 1420-050, PerkinElmer Life Science, Turku, Finland).

### 4.6. Tyrosinase Activity Assay

For measurement of the intracellular tyrosinase activity, we slightly modified a previously described method [23]. Melanoblasts were seeded at 1 × 10^4^ cells per well in six-well plates. After removing the culture medium, the cells were washed with PBS and lysed with 10% Triton X-100 (Sigma Aldrich). Cells were then harvested, centrifuged at 15,000 rpm for 15 min, and the supernatant extracted. We performed a bicinchoninic acid (BCA) assay (Thermo Fisher Scientific, Rockford, IL, USA) on the supernatant to measure protein levels, using bovine serum albumin (BSA) as a standard, adjusted to the same concentration as the sample protein with lysis buffer. L-DOPA (Sigma Aldrich, St. Louise, MO, USA) in sodium phosphate buffer (10 mM) was added, followed by incubation at 37 °C for 30 min. Absorbance was measured at 475 nm, with an ELISA plate reader (Spectrum Analyzer. Victor 1420-050, PerkinElmer Life Science).

### 4.7. RT-qPCR

Total cellular RNA was isolated using 500 μL of TRIzol reagent (Invitrogen). Samples were added to 100 μL of chloroform (Sigma Aldrich), followed by vortexing, and incubation at room temperature for 3 min. After centrifuging at 12,000 rpm at 4 °C for 15 min, the upper supernatant was transferred to empty tubes, and 500 μL of isopropanol was added. After mixing and incubation for at room temperature for 10 min, the solution was centrifuged at 14,000 rpm at 4 °C for 10 min. The supernatant, without pellets, was removed and added to 1 mL of 70% ethanol. After centrifuging at 9000 rpm at 4 °C for 5 min, the supernatant was discarded, and the pellets were dried at room temperature. The dry pellets were added to 20 μL of diethylpyrocarbonate (DEPC)-water and placed on ice.

Total RNA concentration was measured using a Nanodrop device (Thermo Fisher Scientific). A reverse transcriptase (RT) reaction from an Advantage RT-PCR kit (Clontech, Palo Alto, CA, USA) was used to synthesize cDNA from 2 μg of total RNA, following the manufacturer’s protocols. RT-PCR primers were purchased from Bioneer (Taejon, Korea). Tyrosinase and tyrosinase-related protein 2 (TRP-2) were quantified, and the corresponding primer sequences are listed in Table 3. Band images were obtained with Molecular Imager ChemiDoc XRS+ (Bio-Rad, Hercules, CA, USA). ImageJ software (National Institutes of Health, Bethesda, MD, USA) was used for quantitative analysis of the RT-PCR from digitized band pictures.

### 4.8. Western Blotting

Cells were lysed in sample buffer consisting of 2% sodium dodecyl sulfate (SDS), 5% 2-mercaptoethanol, 10% glycerol, and 0.1 mg/mL bromophenol blue in Tris–HCl, pH 6.8, by boiling at 100 °C for 5 min. A BCA assay was performed, with BSA as a standard, to ensure loading of identical protein amounts in the dodecyl sulfate-polyacrylamide gel electrophoresis (SDS-PAGE). Then, 30 μg of the lysed protein was electrophorized by 10% SDS-PAGE and transferred onto a nitrocellulose membrane. The membranes were blocked with 5% skim milk and washed trice in Tris-buffered saline-Tween 20 (TBS-T) for 15 min. Membranes were then incubated with primary antibodies for β-actin (Sigma Aldrich), tyrosinase, tyrosinase-related protein 1 (TRP-1), microphthalmia-associated transcription factor (MITF) (Santa Cruz, CA, USA), extracellular signal-regulated kinase (ERK), p-ERK, CREB, p-CREB, p-p38, phosphorylated stress-activated protein kinase/c-Jun N-terminal kinase (p-SAPK/JNK) (Cell Signaling) diluted in TBS-T containing 5% BSA, 0.05% sodium azide at 4 °C overnight. After washing with TBS-T, the membranes were incubated with horseradish peroxidase-conjugated secondary antibodies, such as anti-rabbit, anti-mouse (Cell Signaling) and anti-goat (Santa Cruz) immunoglobulin G diluted in 5% skim milk in TBS-T. Band images were obtained by using an electrogenerated chemiluminescence system (Thermo Fisher Scientific) and Molecular Imager ChemiDoc XRS+ (Bio-Rad, Hercules, CA, USA). ImageJ software (National Institutes of Health, Bethesda, MD, USA) allowed quantitative analysis of western blotting using digitized band images.

### 4.9. Immunohistochemistry

For immunohistochemistry analysis, coverslips were placed in a 24-well plate, and then cells were seeded directly on the coverslips. After exposure to ELF-EMF, cells were cultured on a coverslip then fixed by incubation in 4% paraformaldehyde at 4 °C for 20 min. Fixed cells were incubated with anti-HMB45 and anti-Melan-A (Abcam, Cambridge, UK, at 1:1000 dilution). Localization of HMB45 and Melan-A was determined using an avidin-immunoalkaline phosphatase method, with vector red as the red chromogen product. Relative staining intensity was scored arbitrarily according to intensities in a light microscopy image, as follows: no or weak staining (−), low intensity (+), moderate intensity (++), and strong intensity (+++). The staining intensity was analyzed with ImageJ software (National Institutes of Health) for the quantitative immunohistochemistry analysis.

### 4.10. Statistical Analysis

Results are reported as the mean ± standard error (SE), and each experiment was repeated at least in triplicate. Data were analyzed by one-way analysis of variance (ANOVA) and Student’s *t*-test. Difference between means was considered significant when *p* < 0.05 (* *p* < 0.05, ** *p* < 0.01). Graphical representations were produced with Sigmaplot 2001 software (Systat Software Inc., San Jose, CA, USA) 

## 5. Conclusions

**Figure 10:**
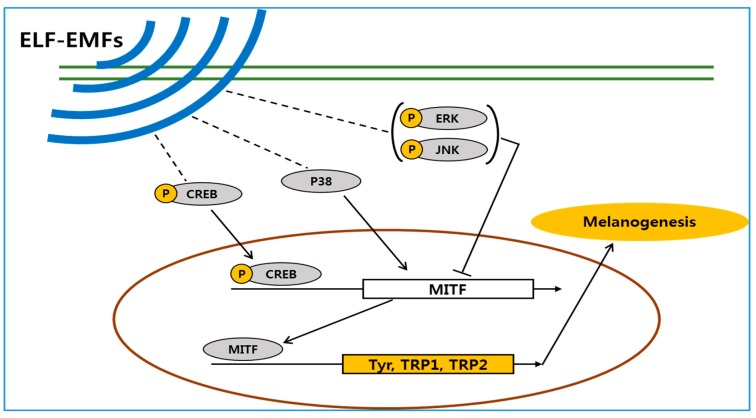
The proposed mechanism by which an extremely low frequency electromagnetic field (ELF-EMF) induces melanin biosynthesis. Schematic shows that ELF-EMFs increases expression of microphthalmia-associated transcription factor (MITF) in human melanocytes. (p-CREB: phosphorylated cAMP response element-binding protein, p38: protein 38kDa, p-JNK: phosphorylated c-Jun N-terminal kinase, p-ERK: phosphorylated extracellular signal-regulated protein kinase, MITF microphthalmia-associated transcription factor, Tyr: tyrosinase, TRP: tyrosinase-related protein)

We have demonstrated that ELF-EMFs can induce melanogenesis in melanocytes. Exposure to ELF-EMFs was associated with increases in the level of p-CREB and p-p38, also inhibited of phosphorylated extracellular signal-regulated protein kinase and phosphorylated stress-activated protein kinase/c-Jun N-terminal kinase. As a results, MITF was upregulated and it’s upregulation enhances the expression of the melanogenesis-related genes tyrosinase, TRP-1, and TRP-2, and stimulates melanin synthesis at 50 Hz (Figure 10).

EMFs are non-invasive and non-toxic, but they interact with biological systems and have health effects. The results of this work may be applicable for gray hair treatment or therapeutically, for inducing repigmentation in the skin of vitiligo patients.

Future studies will focus on EMF-induced melanogenesis in a three-dimensional culture model and in vivo.

## Figures and Tables

**Table 1:** The number of dendritic and multipolar cells (*n* = 4). (MSH: Melanocyte-stimulating hormone).

Dendritic Cell Number	Control	MSH	30 Hz	50 Hz	60 Hz	75 Hz
multipolar morphology	9 ± 3	16 ± 3	11 ± 4	25 ± 4	24 ± 3	18 ± 4

**Table 2:** Relative staining intensity score for HMB45 and Melan-A.

Marker	Control	MSH	30 Hz	50 Hz	60 Hz	75 Hz
HMB45	+	++	+	+++	+++	++
Melan-A	+	++	+	+++	+++	++

**Table 3:** Primers used for reverse transcription real-time quantitative polymerase chain reaction.

Gene	Upstream Primer Sequence	Downstream Primer Sequence
Glyceraldehyde-3-phosphate dehydrogenase	5′-ACC ACA GTC CAT GCC ATC AC-3′	5′-TCC ACC CTG TTG CTG TA-3′
Tyrosinase	5′-CTC AAA GCA TGC ACA AT-3′	5′-GCC CAG ATC TTT GGA TGA AA-3′
Tyrosinase-related protein 2	5′-TTC GGC AGA ACA TCC ATT CC-3′	5′-TTG GCA ATT TCA TGC TGT TTC-3′
